# Supplementary material for: Structure, Properties, and Phase Transformations of Water Nanoconfined between Brucite-like Layers: The Role of Wall Surface Polarity
Source: Materials (Basel). 2022 Apr 22;15(9):3043. doi: 10.3390/ma15093043 (PMC9100153; doi:10.3390/ma15093043)
Supplement: Supplementary file 1 [file materials-15-03043-s001.zip › materials-1682268-supplementary.pdf]

Supplementary Materials for

Structure, Properties and Phase Transformations of Water Nanoconfined between Brucite-like Layers: Role of Wall Surface Polarity

Alexey A. Tsukanov[1*], Evgeny V. Shilko[2*] and Mikhail Popov[3]

[1] Center for Computational and Data- Intensive Science and Engineering (CDISE), Skolkovo Institute of Science and Technology (Skoltech), 121205, Moscow, Russia
[2] Institute of Strength Physics and Material Sciences, Siberian Branch of Russian Academy of Sciences (ISPMS SB RAS), 634055, Tomsk, Russia
[3] Technische Universität Berlin, 10623 Berlin, Germany

[*]Corresponding authors: a.tsukanov@skoltech.ru (A.A.T.), shilko@ispms.tsc.ru (E.V.S.)

1. Choice and construction of the model

Table S1. Lattice constants *a* of different divalent layered metal hydroxides $Me^{(II)}(OH)_2$ with brucite-like structure, and relative difference from the lattice constant of the chosen model.

$Me^{(II)}$	*a*, Å	Ref.	COD* ID	Difference from the model, Δ*a*, %
Ni	3.126	[103]	no	-4.3%
Mg	3.142	[104]	1000054	-3.8%
Co	3.173	[105]	1010267	-2.8%
Zn	3.194	[106]	1529590	-2.2%
Fe	3.265	[58]	9002261	–
Mn	3.322	[107]	no	1.7%
Cd	3.495	[108]	no	7.0%
Ca	3.586	[109]	1000045	9.8%

* – Identification number of the compound in the Crystallography Open Database [110,111].

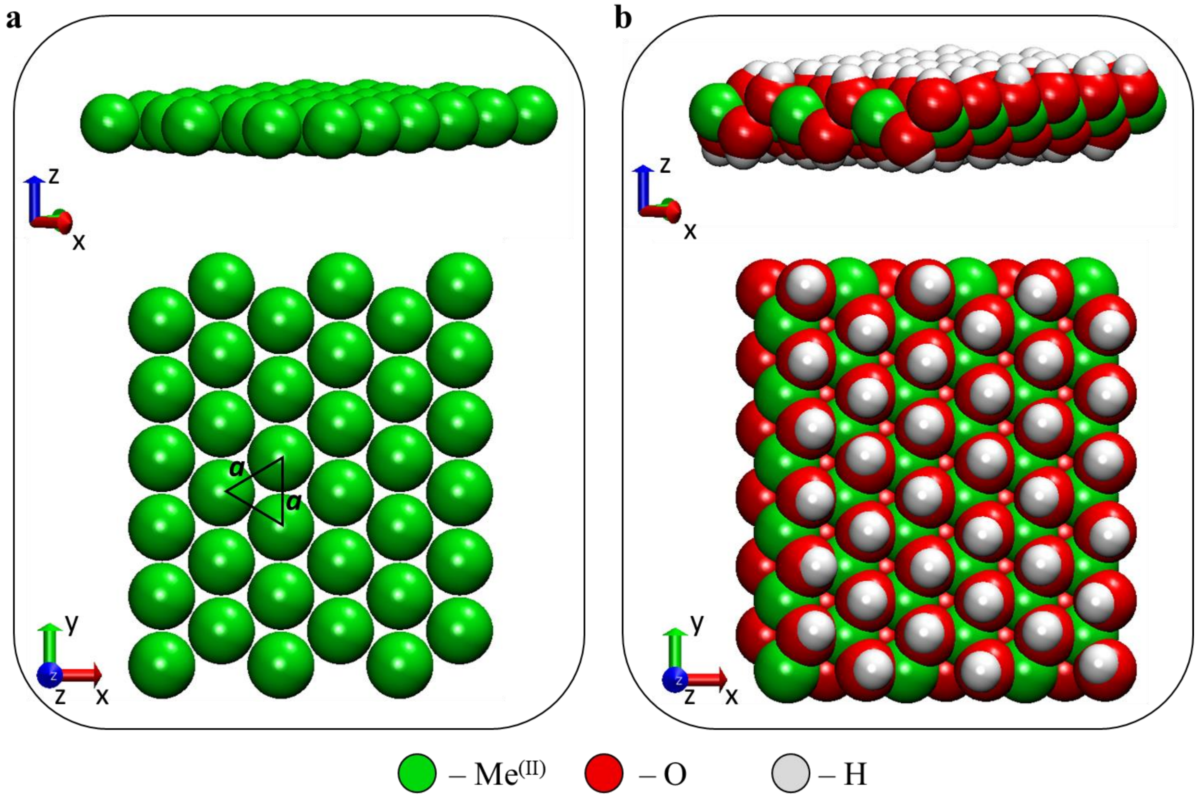

Figure S1. | Structural model of $Me^{(II)}(OH)_2$ nanosheets: ordering of metal atoms within the central plane of the nanosheet (**a**) and full view of the periodical domain of the nanosheet (**b**). Since single mineral nanosheets are considered, only the lattice dimensions in the nanosheet plane are important (*a* = *b*). The lattice constants depend on the ionic radii of the metals, which make up the mineral. Colour code: $Me^{(II)}$ – green, oxygen – red, hydrogen – white.

Table S2. Values of partial atomic charges (PACs) used in the simulations

Atom	Naturally charged wall model PACs, e	Low-charged wall model PACs, e
Divalent metal, $Me^{(II)}$	1.050	0.105
Hydroxyl oxygen, O	–0.950	–0.095
Hydroxyl hydrogen, H	0.425	0.0425
Water oxygen, O (TIP3P)	0.417	0.417
Water hydrogen, H (TIP3P)	–0.834	–0.834

2. Bond and angle parameters

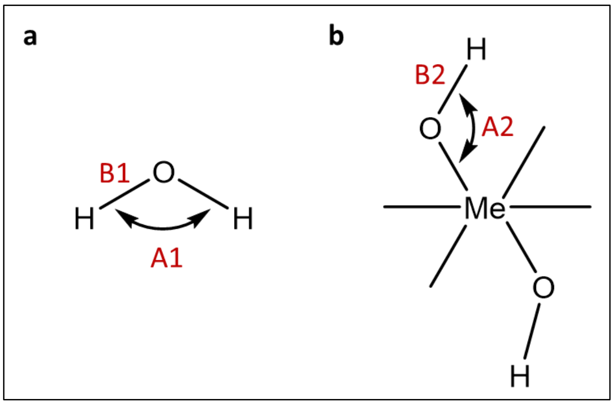

Figure S2. | Covalent bonds and angles explicitly defined in the models of water (**a**) and $Me^{(II)}$ hydroxide (**b**). Corresponding parameters are listed in the Table S3.

Table S3. Factors and equilibrium values of bond length and angles between covalently bonded atoms.

#	Compound	Bond (force field)	k_b, kcal/(mol·Å^2)	b_0, Å
B1	Water	O-H (TIP3P [62])	450.0	0.9572
B2	$Me^{(II)}(OH)_2$	O-H (ClayFF [59])	554.1349	0.9630
**#**	**Compound**	**Angle (force field)**	**k_γ, kcal/(mol·deg^2)**	**γ_0, °**
A1	Water	H-O-H (TIP3P)	30	104.52
A2	$Me^{(II)}(OH)_2$	Me-O-H (ClayFF)	55	109.47

3. Spatial arrangement of water molecules

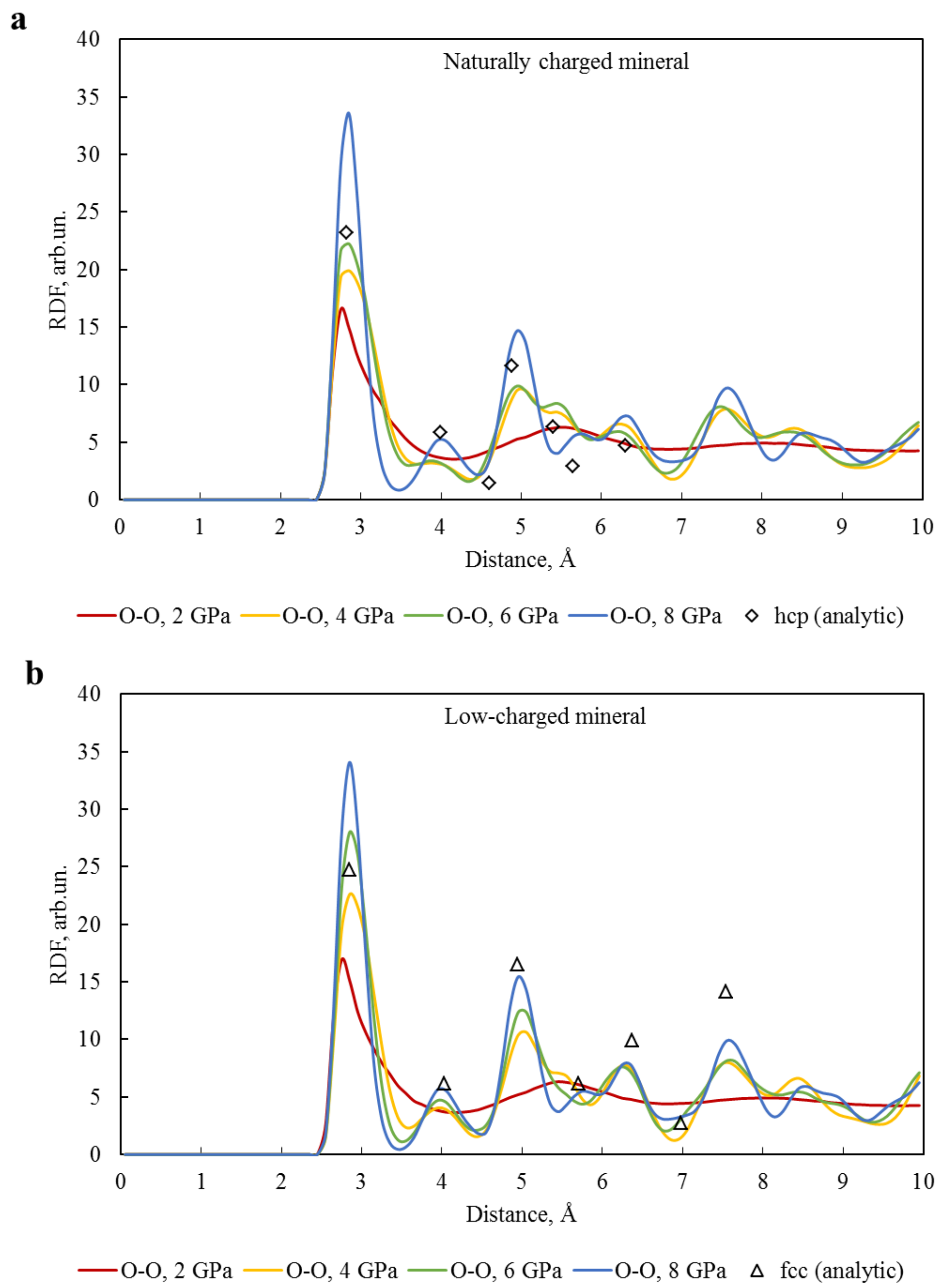

Figure S3. | Oxygen-oxygen radial distribution functions for nanoconfined water in naturally charged
(**a**) and low-charged (**b**) host minerals at different pressures (2, 4, 6 and 8 GPa).

Supplementary Fig.S3 shows radial distribution functions (RDFs) for oxygen-oxygen pairs of the nanoconfined water in hydrophilic (Fig. S3**a**) and hydrophobic (Fig. S3**b**) minerals. One can see that at a pressure of 2 GPa, corresponding to the liquid state of confined water, RDFs are similar for both host minerals (red curves). Note that the absence of sharp peaks and the smooth profiles of these (red) curves are typical for liquid water. RDFs for nanoconfined water in hydrophilic and hydrophobic host minerals at a pressure of 8 GPa are also close to each other (blue curves) since the confined water at this pressure has an fcc-like lattice type in both systems. At the same time, RDFs for the considered systems under intermediate pressure conditions are different (yellow and green

curves at Figs. S3**a** and S3**b**) since nanoconfined water in the hydrophilic host has hcp-like lattice at pressures of 4 and 6 GPa, while water in the hydrophobic host has fcc-like crustal structure. Analytically calculated positions and values of the first 7 peaks of RDF for idealized hcp and fcc lattices are shown by diamonds (on Fig.S3**a**) and triangles (on Fig.S3**b**), respectively. One can see that the positions of the peaks of the curves for nanoconfined water in the hydrophilic host at pressures 4 and 6 GPa (yellow and green) match well with the positions of the analytical markers (diamonds) for idealized hcp lattice with a lattice constant ~ 2.8 Å (the difference is explained by thermal smearing). Similarly, the positions of the peaks of the curves for nanoconfined water in the hydrophilic host (at 8 GPa) and in the hydrophobic host (at 4, 6 and 8 GPa) match well with the positions of the analytical markers (triangles) for idealized fcc lattice with a lattice constant ~ 4.0 Å.

a

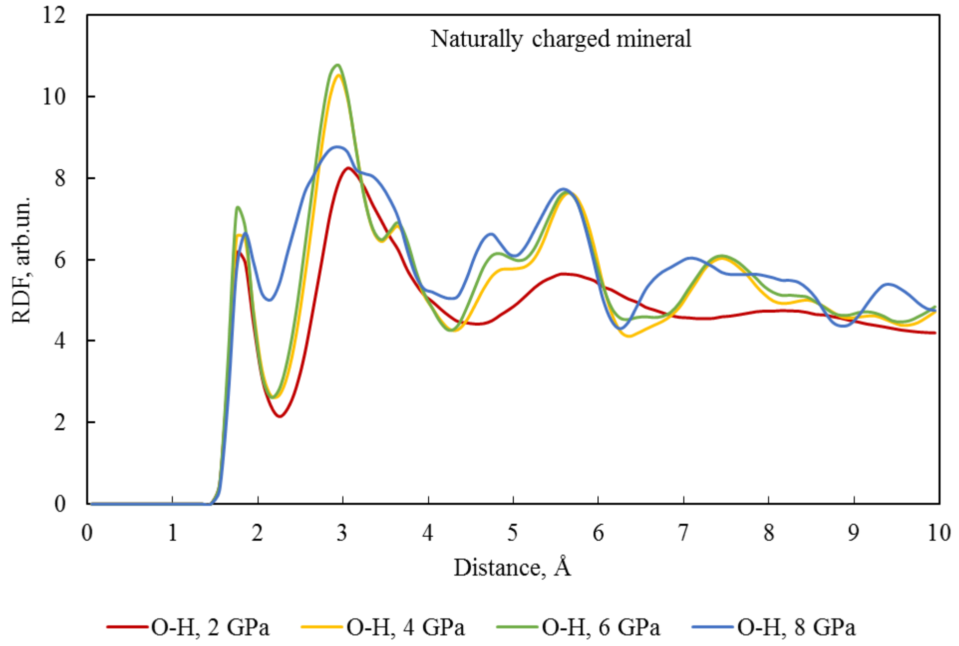

b

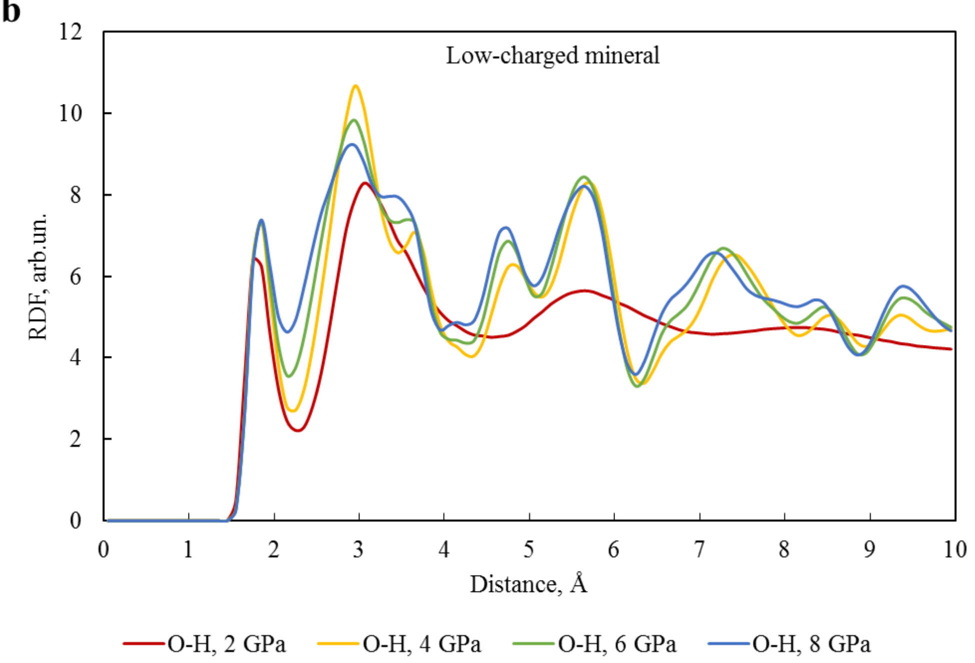

Figure S4. | Hydrogen-oxygen radial distribution functions for nanoconfined water in naturally charged

(**a**) and low-charged (**b**) host minerals at different pressures (2, 4, 6 and 8 GPa).

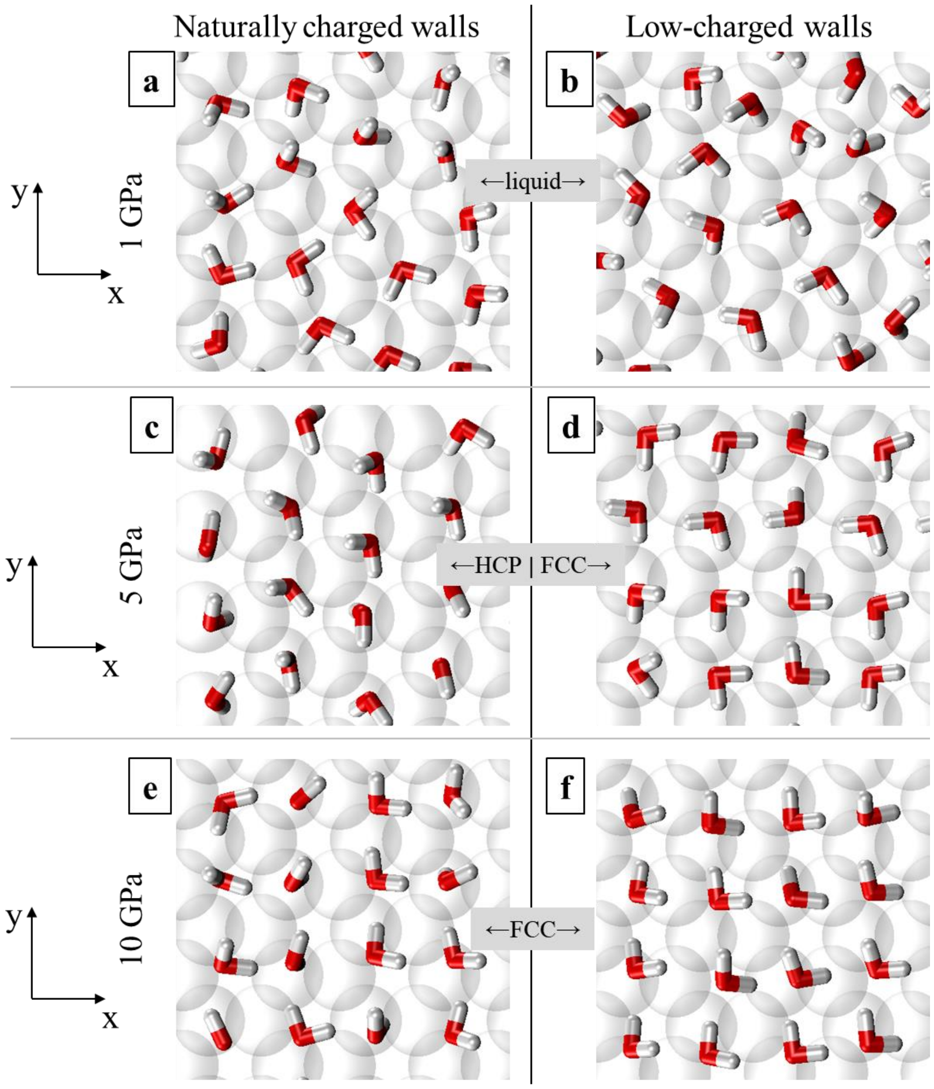

Figure S5. | Views in the plane of the first near-surface layer of water confined in the nanopore at different pressures: p = 1 GPa (**a**, **b**), p = 5 GPa (**c**, **d**) and p = 10 GPa (**e**, **f**). Left column shows representative structure (positions and orientations) of water layer near naturally charged mineral wall, the right one shows the same in case of low-charged mineral. Colours: hydrogen – white, water oxygen – red, mineral oxygen – transparent.

4. Hydrogen bond analysis

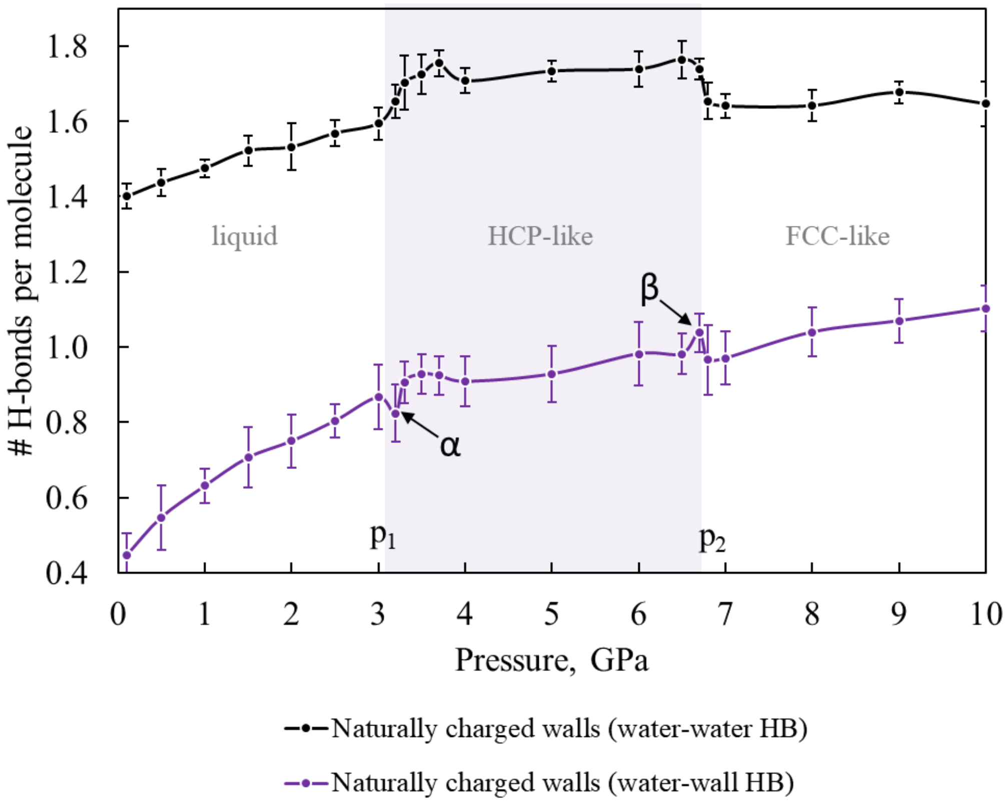

Figure S6. | Pressure dependencies of the number of water-water (per molecule) and water-wall (per surface OH-group) hydrogen bonds in naturally charged system. There are two points α and β of unstable behavior of water-wall H-bonds observed near the phase transition pressures p_1 and p_2, respectively. The general trend of the average water-wall hydrogen bond number is an increase from 0.45 at 0.1 GPa to 1.10 at 10 GPa.

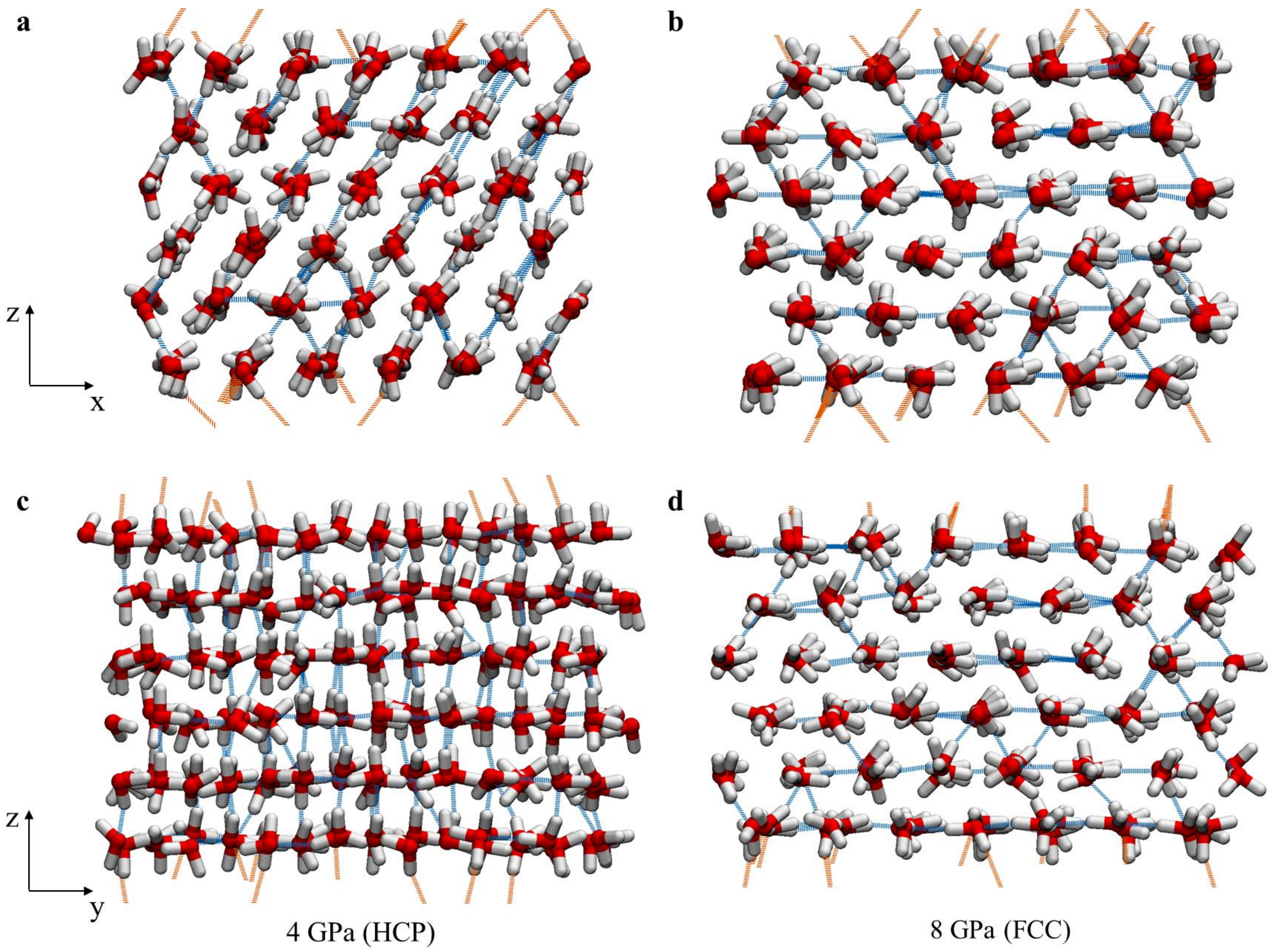

Figure S7. | Hydrogen bonding in the nanopore of the naturally charged system at pressures p = 4 GPa (**a**, **c**) and p = 8 GPa (**b**, **d**). Colors: H-bonds with mineral surface – orange, water-water H-bonds – blue, hydrogen atoms – white, oxygen – red. The near-surface water molecules participate in the formation of hydrogen bonds with the surface of the mineral (orange dashed lines) both as proton donor (line starting at a hydrogen atom) and as proton acceptor (line starting at an oxygen).

5. Crystallization pressure of water in the bulk phase

According to ref.[70], in the temperature range from 273.31 K to 355 K water crystalizes into ice VI, at pressures $p \geq p^*(\mathrm{T})$, where p^* is determined by the following relationship (in MPa):

$$p^*(\mathrm{T}) = 632.4 \times [1 - 1.07476 \times (1 - (\mathrm{T} / 273.31)^{4.6})], \quad (1)$$

For T = 300 and 310 K bulk water crystallizes at

$$p^*(300\ \mathrm{K}) = 996.1\ \mathrm{MPa} \quad (2)$$

and

$$p^*(310\ \mathrm{K}) = 1166.0\ \mathrm{MPa}, \quad (3)$$

respectively.
